# Supervision of a self-driving vehicle unmasks latent sleepiness relative to manually controlled driving

**DOI:** 10.1038/s41598-021-92914-5

**Published:** 2021-09-14

**Authors:** Erin E. Flynn-Evans, Lily R. Wong, Yukiyo Kuriyagawa, Nikhil Gowda, Patrick F. Cravalho, Sean Pradhan, Nathan H. Feick, Nicholas G. Bathurst, Zachary L. Glaros, Theerawit Wilaiprasitporn, Kanika Bansal, Javier O. Garcia, Cassie J. Hilditch

**Affiliations:** 1 Fatigue Countermeasures Laboratory, Human Systems Integration Division, NASA Ames Research Center, Moffett Field, CA USA; 2 Fatigue Countermeasures Laboratory, San José State University, Moffett Field, CA USA; 3 College of Industrial Technology, Nihon University, Chiba, Japan; 4 School of Business Administration, Menlo College, Atherton, CA USA; 5 School of Information Science and Technology, Vidyasirimedhi Institute of Science and Technology, Rayong, Thailand; 6 Army Research Laboratory, U.S. CCDC, Aberdeen Proving Ground, MD USA; 7 Department of Biomedical Engineering, Columbia University, New York, NY USA

**Keywords:** Circadian rhythms and sleep, Cognitive neuroscience, Human behaviour

## Abstract

Human error has been implicated as a causal factor in a large proportion of road accidents. Automated driving systems purport to mitigate this risk, but self-driving systems that allow a driver to entirely disengage from the driving task also require the driver to monitor the environment and take control when necessary. Given that sleep loss impairs monitoring performance and there is a high prevalence of sleep deficiency in modern society, we hypothesized that supervising a self-driving vehicle would unmask latent sleepiness compared to manually controlled driving among individuals following their typical sleep schedules. We found that participants felt sleepier, had more involuntary transitions to sleep, had slower reaction times and more attentional failures, and showed substantial modifications in brain synchronization during and following an autonomous drive compared to a manually controlled drive. Our findings suggest that the introduction of partial self-driving capabilities in vehicles has the potential to paradoxically increase accident risk.

## Introduction

Human error has been implicated as a causal factor in a large proportion of motor vehicle crashesx^1^, with drowsy driving accounting for up to 20% of accidents^2–4^. Technological advances, such as automated braking and blind-spot monitoring, have mitigated some of this risk. For instance, vehicle manufacturers have developed Advanced Driver Assistance Systems (ADAS) to mitigate the repercussions of drowsy driving. Crash avoidance features, such as forward collision warning systems, automatic emergency braking, and blind spot monitoring systems, have all been shown to reduce the risk of an accident by 27–56% relative to vehicles without such features^5,6^. However, other recent advances, such as the introduction of self-driving vehicle capabilities, eliminate all driving tasks except for monitoring of the environment and automation. Vehicles with such capabilities allow the driver to enable and disable the self-driving system^7^, but require the driver to be prepared to retake control of the vehicle at any time. As the driver’s role shifts from one of active engagement to passive monitoring, partially autonomous systems may introduce new safety concerns.

Monitoring tasks that require sustained attention are particularly difficult for humans to perform, especially when they are sleep deprived^8,9^. Nearly 33% of the United States (US) population sleeps six or fewer hours per night^10^, and up to 90% of workers commute by private vehicle^11^. Therefore, it is not surprising that many people drive when sleep deprived. These factors raise the concern that requiring individuals to supervise and vigilantly respond to hazards when a vehicle is in self-driving mode may inadvertently increase risk by reducing the ability of the driver to respond when needed. Given the 1) high prevalence of sleep deficiency in the US; 2) increasing autonomous capabilities in vehicles; and 3) difficulty that humans experience with monitoring tasks that require sustained attention; we hypothesized that supervision of a self-driving vehicle by people following their typical sleep schedules would unmask latent sleepiness relative to manually controlled driving.

## Results

We evaluated 59 individuals in three randomized, within-subjects simulated driving studies. In Study 1, we investigated whether participants’ self-reported alertness, objective sleepiness and brain coordination, and vigilant attention would differ when supervising a self-driving vehicle compared to manually controlled driving. Study 2 served as a replication to validate our findings. In order to expand the generalizability of the study, participants in Study 3 completed both drives while listening to a music playlist of their choice.

During the laboratory visit, participants completed two approximately 48-min simulated driving sessions in a randomized order: one where they had full control of the vehicle dynamics (i.e., steering, acceleration, brakes); and one in which they had no control of the vehicle dynamics (self-driving/autonomous) but were instructed to keep their hands on the steering wheel while monitoring the vehicle. During both drives, participants wore electrodes to monitor slow rolling eye movements (SREMs), which are characteristic of transitioning to sleep^12^, and neural activity (Fz, Cz, Pz, Oz). Participants rated their sleepiness using the Karolinska Sleepiness Scale (KSS) and completed a five-minute psychomotor vigilance task (PVT) to evaluate vigilant attention immediately following each drive. Demographic characteristics of the participants are presented in Table 1.

**Table 1:** Participant demographics.

	Study 1* M *(± *SD*)	Study 2 (Replication) *M* (± *SD*)	Study 3 (Music) *M* (± *SD*)
*n*	19	20	20
Age (years)	33.0 (10.4)	35.9 (15.3)	34.1 (15.1)
Sleep duration (h)*	5.9 (0.9)	5.9 (1.0)	6.1 (0.9)
Habitual bedtime	00:46 (1:35)	0:07 (1:20)	0:22 (1:22)
Habitual waketime	7:49 (1:33)	7:12 (1:22)	7:20 (1:12)
PSQI	5.4 (3.9)	5.0 (2.3)	6.4 (2.7)
FSS	29.2 (7.4)	31.2 (10.3)	30.0 (8.5)
MEQ	50.0 (12.4)	52.5 (8.4)	50.8 (9.4)
Daily caffeine (cups)	1.5 (1.2)^†^	1.2 (1.4)	1.3 (1.5)
Years licensed	15.6 (10.3)^†^	18.8 (15.0)	17.4 (13.5)
Weekly drive time (h)	6.4 (4.6)^†^	10.3 (7.4)	8.2 (6.2)
Commute time (m)	34.5 (33.0)^†^	46.7 (35.5)	42.5 (31.9)
	*n* (%)	*n* (%)	*n* (%)
Male	11 (58)	10 (50)	11 (55)

*As measured by actigraphy during the two weeks prior to the study; ^†^*n* = 13 for these questions, as a questionnaire with these items was sent retrospectively to participants in Study 1; Commute time = one way; PSQI = Pittsburgh Sleep Quality Index; FSS = Fatigue Severity Score; MEQ = Morningness-Eveningness Questionnaire; h = hours; m = minutes; *M* = mean; *SD* = standard deviation.

### Supervision of a self-driving vehicle unmasks objective sleepiness relative to manually controlled driving

**Figure 1:**
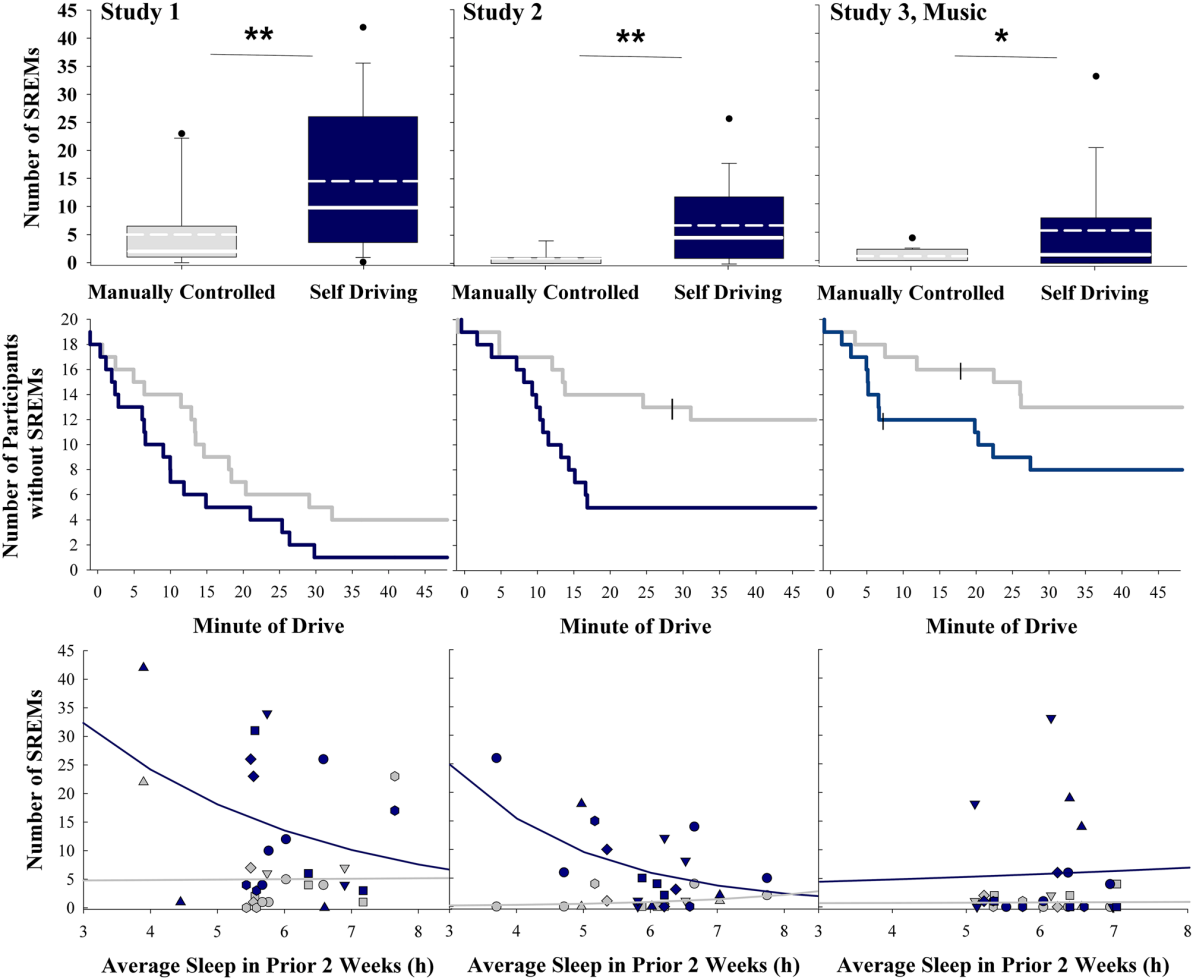
Slow rolling eye movement (SREM) characteristics during the manually controlled and self-driving conditions for each study. A greater number of SREMs indicates higher sleepiness. Top row = number of SREMs by condition and study. For each box, the solid white line indicates the median, the dashed white line indicates the mean, and the bottom and top boundaries of the box indicate the 25th and 75th percentiles, respectively. The bottom and top whiskers indicate the 10th and 90th percentiles, respectively (Study 1 *n* = 17; Study 2 *n* = 19; Study 3 *n* = 18). Middle row = Survival plots showing time to first SREM by condition and study. Blue = self-driving, gray = manually controlled, hatched lines indicate the time that individual participants were censored due to premature termination of the drive (Study 1 *n* = 19; Study 2 *n* = 20; Study 3 *n* = 20). Bottom row = Number of SREMs plotted by sleep duration in the prior two weeks by condition and study. Fit lines represent the negative binomial regression. Blue = self-driving, gray = manually controlled (Study 1 *n* = 17; Study 2 *n* = 19; Study 3 *n* = 18). * = *p* < 0.05; ** = *p* < 0.01.

Participants experienced more slow rolling eye movements (SREMs), which are signatures of the transition from wake to sleep, in the self-driving (*M* ± *SD* Study 1 = 14.47 ± 13.32; Study 2 = 6.89 ± 7.25; Study 3 = 5.78 ± 9.27) compared to the manually controlled driving condition (*M* ± *SD* Study 1 = 5.00 ± 7.02, *χ*^2^[1] = 14.05, *p* < 0.001; Study 2 = 0.84 ± 1.38, *χ*^2^[1] = 30.00, *p* < 0.001; Study 3 = 0.78 ± 1.17, *χ*^2^[1] = 16.53, *p* < 0.0001), irrespective of whether they drove with our without listening to music (Fig. 1, top row). The effect sizes for each of these comparisons were moderate to strong (*Hedge’s g*_*adj*_ Study 1 = 0.79; Study 2 = 1.05; Study 3 = 0.68). In addition, participants experienced their first SREM sooner in the self-driving compared to the manually controlled driving condition (*log-rank test* Study 1 *p* = 0.02; Study 2 *p* = 0.005; Fig. 1, middle row), although this difference was not statistically significant for the music study (Study 3 *p* = 0.11). Participants with shorter habitual sleep duration had more SREMs for the self-driving condition in Study 2 (self-driving *log*[*b*] = -0.48, *log*[95% CI = -0.87, -0.09], *p* = 0.02; manually controlled *log*[*b*] = 0.48, *log*[95% CI = -0.43, 1.34], *p* = 0.30; Fig. 1, bottom row), with a similar, but non-significant trend in Study 1 (self-driving *log*[*b*] = -0.29, *log*[95% CI = -0.74, 0.16], *p* = 0.20; manual *log*[b] = 0.02, *log*[95% CI = -1.34, 1.37], *p* = 0.98). SREMs were not associated with prior sleep history in either driving condition in the music study (Study 3 self-driving *log*[*b*] = 0.09, *log*[95% CI = -0.81, 0.99], *p* = 0.85; manual *log*[b] = 0.06, *log*[95% CI = -1.22, 1.34], *p* = 0.93; Fig. 1, bottom row, far-right panel), suggesting that music may influence arousal.

### Supervision of a self-driving vehicle unmasks subjective sleepiness relative to manually controlled driving

**Figure 2:**
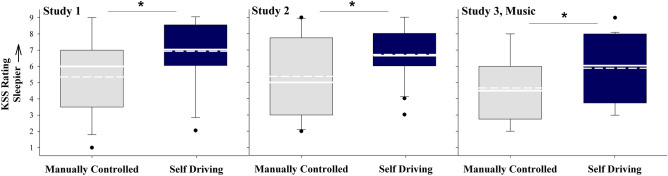
Self-reported sleepiness following the manually controlled and self-driving conditions for each study. Higher Karolinska Sleepiness Scale (KSS) scores indicate higher sleepiness. For each box, the solid white line indicates the median, the dashed white line indicates the mean, and the bottom and top boundaries of the box indicate the 25th and 75th percentiles, respectively. The bottom and top whiskers indicate the 10th and 90th percentiles, respectively (Study 1 *n* = 17; Study 2 *n* = 19; Study 3 *n* = 19). * = *p* < .05.

Participants rated themselves as significantly sleepier following the self-driving condition (*M* ± *SD* Study 1 = 6.88 ± 2.06; Study 2 = 6.63 ± 1.77; Study 3 = 5.89 ± 1.97) relative to the manually controlled drive (*M* ± *SD, Wilcoxon signed-rank* Study 1 = 5.35 ± 2.42, *V* = 33, *p* = 0.04; Study 2 = 5.45 ± 2.35, *V* = 41, *p* = 0.049; Study 3 = 4.74 ± 2.02, *V* = 38.5, *p* = 0.04; Fig. 2), irrespective of whether or not they listened to music while driving. The effect sizes for each of these comparisons were moderate (*Hedge’s g*_*adj*_ Study 1 = 0.61; Study 2 = 0.51; Study 3 = 0.52).

### Supervision of a self-driving vehicle is associated with slowed reaction times and increased lapses of attention relative to manually controlled driving while driving without music

**Figure 3:**
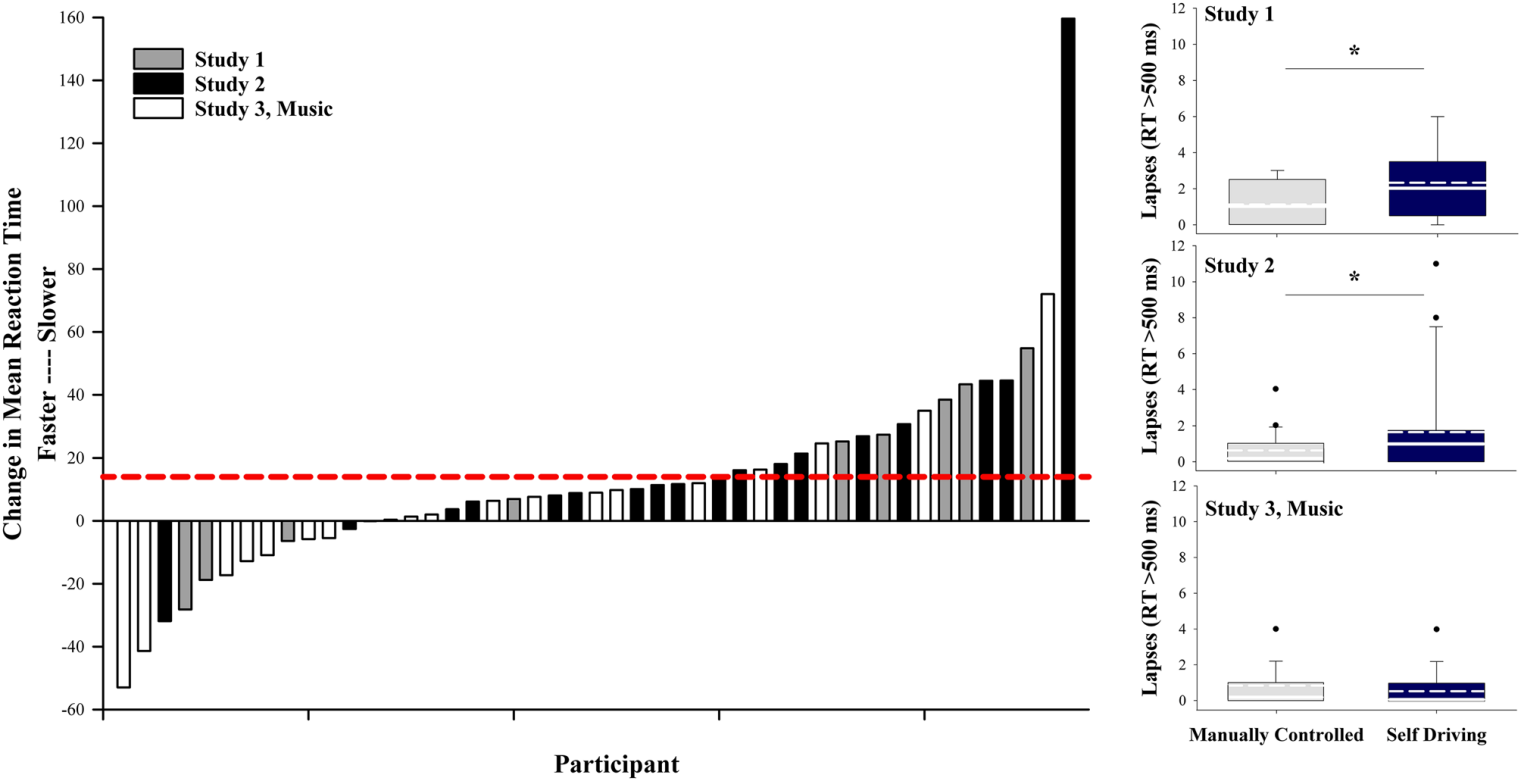
Mean reaction time and lapses of attention following the manually controlled and self-driving conditions. Left panel: Change in mean reaction time following the self-driving compared to the manual-driving condition by individual participant. Higher values indicate slower reaction times. The red dashed line indicates reaction time slowing by 15 ms, which is the equivalent increase in reaction time observed in those with a blood alcohol concentration of 0.5 g/L relative to placebo. Right panel: Lapses (reaction time [RT] > 500 ms) following the manually controlled and self-driving conditions. Higher values indicate more lapses in attention. For each box, the solid white line indicates the median, the dashed white line indicates the mean, and the bottom and top boundaries of the box indicate the 25th and 75th percentiles, respectively. The bottom and top whiskers indicate the 10th and 90th percentiles, respectively (Study 1 *n* = 9; Study 2 *n* = 19; Study 3 *n* = 19). * = *p* < .05.

Participants completed a five-minute psychomotor vigilance task (PVT), a simple reaction time test measuring vigilant attention, following each drive. The majority of participants (72%) had a slower reaction time following the self-driving condition relative to the manually controlled drive (Fig. 3). Furthermore, in 36% of participants, average reaction times slowed by at least 15 ms, which is the equivalent average increase in reaction time observed in those with a blood alcohol concentration of 0.5 g/L relative to placebo^13^. Participants had slower mean reaction times following the self-driving condition (*M* ± *SD* Study 1 = 318.17 ± 48.48; Study 2 = 294.28 ± 53.41) compared to the manually controlled drive (*M* ± *SD* Study 1 = 302.30 ± 34.81, *b* = -15.87, *p* = 0.14; Study 2 = 272.69 ± 34.97, *b* = -21.59, *p* = 0.03) in the non-music driving studies. However, this difference was not significantly different in Study 1, likely due to our limited sample size in that analysis (*n* = 9). Notably, the differences in mean reaction times between participants in Study 1 and Study 2 likely result from individual differences in performance between these groups as has been documented previously^8^. There was no statistically significant difference in mean reaction time between conditions in the music study (*M* ± *SD* Study 3, self-driving = 265.39 ± 35.05; manually controlled = 262.79 ± 27.80, *b* = -2.60 *p* = 0.67). Participants had significantly more lapses (reaction times > 500 ms) following the self-driving condition (*M* ± *SD* Study 1 = 2.33 ± 1.94; Study 2 = 1.68 ± 2.93) compared to the manually controlled drive (*M* ± *SD* Study 1 = 1.00 ± 1.22, *χ*^2^[1] = 9.13, *p* = 0.003; Study 2 = 0.63 ± 1.01, *χ*^2^[1] = 7.80, *p* = 0.005) in the non-music driving conditions. The effect sizes for the differences in lapses in the non-music driving studies were moderate (Hedge’s g_*adj*_ Study 1 = 0.58; Study 2 = 0.43). There was no difference in lapses between conditions in the music study (*M* ± *SD* Study 3, self-driving = 0.53 ± 1.02; manually controlled = 0.79 ± 1.03, *χ*^2^[1] = 0.64, *p* = 0.42).

### Supervision of a self-driving vehicle is associated with decreases in neural synchronization relative to manually controlled driving

Electroencephalography (EEG) was continuously collected across the midline of the scalp in four electrodes (Fz, Cz, Pz, Oz) during each driving task. Due to the expected differences in eye movements between the drives, confounds due to eye movements and blinks that could potentially contaminate the EEG collected at the scalp were removed. After this preprocessing step, two measures of neural activity were estimated: (i) the power spectral density, and (ii) *synchronization,* estimated using a novel connectivity approach called the binarized derivative method (BDM)^14^, which is an estimate of the bias of power fluctuations to increase and decrease in concert across the channels.

**Figure 4:**
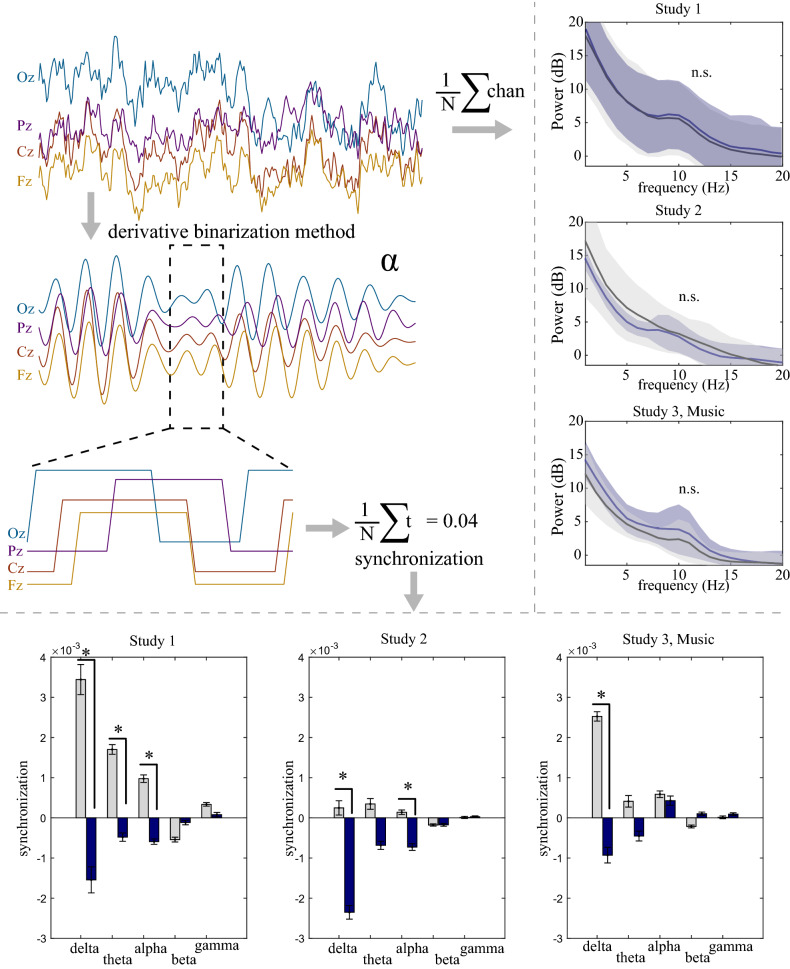
Power and synchronization during the manually controlled and self-driving conditions. Sample participant fluctuations for approximately 1.5 s of EEG data for the midline electrodes and trajectories to the two different analyses: (i) Right, the average power across frequencies for both the manually controlled and self-driving conditions; and (ii) Bottom, the bandpass filtered and binarized segments of the binarized derivative method (BDM), as shown for a small segment of the EEG, where the binarized signals are then summed and averaged to estimate the *synchronization* of the signals. Right panel: Mean power spectral density across frequencies for both driving conditions for Studies 1–3. Blue = self-driving, gray = manually controlled. Semi-transparent shading surrounding this average indicates the standard deviation across participants. Lower panel: Mean synchronization for 5 frequency bands (delta: 1–3 Hz, theta: 4–7 Hz, alpha: 8–12 Hz, beta: 13–25 Hz, gamma: 26–40 Hz) of both driving conditions for Studies 1–3 (Study 1 *n* = 17; Study 2 *n* = 20; Study 3 *n* = 18). Error bars indicate standard error of the mean (SEM) across participants. * = *p* < .05.

To determine whether the driving conditions differed in overall power or synchronization, we completed several statistical comparisons. First, it is well established that fluctuations in power reflect a variety of cognitive functions and states^15^; however, paired-sample *t*-tests for each frequency within each study did not reveal any consistent difference between driving conditions (*p* > 0.05). As shown in the right panel in Fig. 4, mean power in each study appeared to change as a function of frequency, but none revealed a consistent increase or decrease in power for either driving condition. Next, we sought to determine whether synchronization within each frequency band tracked any difference between the driving conditions, as connectivity within the brain has been shown to mark different functions and states, similar (but unique) to power^16,17^. For each study and frequency band (delta, theta, alpha, beta, gamma), we employed a linear mixed model and found significant main effects of driving condition (manually controlled, self-driving) primarily within the delta and alpha bands (Fig. 4, bottom panel). Participants had significantly lower synchronization across the brain within the delta band during the self-driving condition compared to the manually controlled drive in each study (Study 1: *b* = 4.9 × 10^–3^, *p* < 0.001; Study 2: *b* = 2.6 × 10^–3^, *p* = 0.003; Study 3: *b* = 3.5 × 10^–3^, *p* = 0.002). Within the alpha band, synchronization was also lower for the self-driving condition compared to the manually controlled drive in the non-music studies (Study 1: *b* = 1.6 × 10^–3^, *p* < 0.001; Study 2: *b* = 8.6 × 10^–4^, *p* = 0.025). The only other frequency band that had a significant effect was within the theta band for Study 1, where the self-driving condition elicited less synchrony than the manually controlled drive (*b* = 2.2 × 10^–3^, *p* = 0.002). For the synchronization results in the lower panel of Fig. 4, we also performed paired-samples *t*-tests to determine the specific effects within each frequency band. Results were identical to the linear modelling effects, where there was a significant decrease in synchronization within the delta band between driving conditions in all studies (Study 1 *t*[16] = -3.91, *p* = 0.001; Study 2 *t*[19﻿] = -3.09, *p* = 0.006; Study 3 *t*[17] = -3.15, *p* = 0.006). Similarly, within the alpha band, there was also significantly lower synchronization during the self-driving condition in the non-music studies (Study 1: *t*[16] = -4.43, *p* < 0.001; Study 2: *t*[19] = -2.28, *p* = 0.034), and Study 1 retained the significant effect within the theta band synchronization, (*t*[16] = -3.06, *p* = 0.008).

## Discussion

We found that participants had more SREMs, had their first SREM sooner, reported feeling sleepier, and had less coordinated neural behavior while supervising a self-driving vehicle relative to manually controlled driving. This was accompanied by a slowed reaction time and greater number of lapses on the PVT following the self-driving condition. Listening to music while driving attenuated many of these effects, but participants in the music study (Study 3) still had more SREMs during, and reported feeling sleepier after, the self-driving condition compared to manually controlled driving. Given the high prevalence of sleep deficiency worldwide, our findings suggest that partially automated vehicles have the potential to increase operator error because drivers are not able to sustain vigilant attention while supervising a self-driving vehicle when following their typical sleep schedules.

Our findings have important implications for drivers on the road today. Self-driving vehicle capabilities are divided into six levels ranging from level 0 (no automation) where the driver performs all driving tasks, to level 5 (full automation) where the Automated Driving System (ADS) on the vehicle is capable of performing all the driving tasks in any situation^18^. Fully autonomous vehicles (level 5) will obviate the need for human monitoring and input, making the state of the human passenger irrelevant to the performance of the vehicle. However, the partial self-driving capabilities currently available in vehicles are classified as level 2 or 3, which requires the driver to continuously monitor the driving environment and prove readiness (e.g., maintaining hands on the wheel or eyes on the road) in order to be ready to take control when needed (e.g., in situations where the vehicle loses lane guides)^19^. Although we did not require the driver to take control of the vehicle during the self-driving condition, we instructed our participants to keep their hands on the wheel and monitor the driving environment. However, other iterations of Level 2 and Level 3 automation allow drivers to remove their hands from the wheel for comfort as long as they maintain situational awareness and are prepared to take over the vehicle if necessary. It is possible that removing one’s hands from the wheel could further reduce engagement and potentially lead to faster expression of sleepiness. Further research is needed to understand how different modes of engagement may influence the expression of sleepiness among drivers.

Our findings suggest that monitoring the driving automation system exploits the driver’s cognitive weaknesses. Several accidents have occurred while such vehicles have been in self-driving mode^20,21^. Indeed, even expertise in automation does not inoculate a driver from cognitive failures, as a fatal accident occurred when a self-driving vehicle was under the supervision of a professional safety driver^22^. Although fatigue was not identified as a causal factor in that incident, our findings suggest that even moderate sleep loss, such as that experienced by millions of people every day, increases the expression of sleepiness when individuals supervise self-driving vehicles relative to manually controlled driving. In addition, news reports have shown drivers falling asleep while using level 2 automation on the highway^23,24^, which is consistent with our finding that individuals have difficulty sustaining wakefulness while supervising a self-driving vehicle.

Our findings confirm and extend prior research by providing evidence that monitoring performance is impaired while supervising a self-driving vehicle. In a similar study on a test track, Kundinger et al. found that sleepiness increased over the course of a 45-min drive in a vehicle with Level 2 automation^25^. In a simulated driving study, Saxby et al.^26^ reported that drivers whose only task was to monitor and respond to automation failures had slower braking and steering responses to a sudden event and were more likely to crash into a van compared to manually controlled driving. Similarly, Greenlee et al.^27^ evaluated reaction time during a simulated self-driving vehicle supervision task and found that reaction time slowed over the course of a 40-min drive. A recent consumer reporting study evaluated lane deviations and system disengagement (i.e., return of vehicle control to the driver) for five vehicles with partially-autonomous features and found that in a simple drive including hills and curves, each vehicle experienced situations that required driver input^28^. When coupled with our findings, these studies suggest that monitoring of level 2 automation is necessary, and the consequences of supervising a self-driving vehicle while sleepy are likely to impair driving performance and worsen with time on task. Supervision of autonomous vehicles has also been shown to increase the likelihood that drivers will engage in non-driving tasks, delay reaction time to critical situations, and increase risk of collision^29–31^. In addition, distraction propensity increases among drowsy individuals, which could be hypothesized to worsen^32^ or paradoxically improve situational awareness^33,34^. In our study, it is unlikely that increased distraction rates could explain our findings, because participants were required to maintain the same driving position in both conditions for all three studies. Furthermore, objective and subjective expression of sleepiness was similar when individuals drove with and without listening to music.

Although our findings suggest that drowsy individuals have difficulty monitoring autonomous systems, it is important to note that manually driving a vehicle while drowsy is associated with increased risk of an accident compared to driving while rested^35,36^. Although a drowsy individual can successfully complete a manually controlled drive without incident^37^, sleep deficiency increases the risk of an accident by creating a layer of vulnerability that slows reaction time and impairs decision making. This vulnerability has the potential to interact with other factors that we did not evaluate in our study, such as inclement weather, traffic density, frequency of stops and lane changes or terrain changes (e.g., hills and curves), to lead to an accident. Consistent with this notion, in our study, many participants had SREMs in the manual driving condition, suggesting that individuals are not immune to the effects of sleep loss when completing an engaging task. Importantly, the impairment that we observed during the self-driving condition was much worse than what we observed during the manual drive. We found that the majority of individuals across all three studies had slower reaction times in the self-driving condition. The average change in reaction time between the manual and self-driving conditions in Study 1 and Study 2 was > 15 ms, which is equivalent to the change in reaction time observed among individuals with a blood alcohol concentration of 0.5 g/L relative to placebo^13^. In addition, participants experienced one additional lapse of attention on the PVT following the self-driving condition, which was double the rate of lapses following the manually controlled driving condition.

Listening to music while driving was not associated with differences in reaction time or lapses between the conditions. Prior studies have demonstrated that listening to self-selected music increases mental effort, which can enhance some aspects of driving performance^38^. For example, Ünal et al. found that drivers who listened to music while completing a manually controlled drive exhibited increased mental effort compared to those who drove without listening to music. However, music may influence arousal or modify attentional resources during low workload activities such as during supervision of a self-driving vehicle^39^. Importantly, our findings suggest that music is not a suitable countermeasure to improve alertness while supervising a self-driving vehicle, as music modified, but did not fully suppress the expression of objective and subjective indicators of sleepiness.

We found that individuals experienced more SREMs in the self-driving condition relative to the manual condition, even while listening to music. The drive that our participants completed was relatively short, occurred during the day, and our participants followed their normal sleep schedules and were moderately sleep deprived. It is likely that the act of manually controlling the vehicle suppressed the expression of SREMs for some sleepy individuals, potentially through the temporary recruitment of attentional resources from other brain regions. That is, the manually controlled driving task requires more complex (i.e., physical and mental) coordination to complete relative to the simple monitoring behavior required for supervising the self-driving condition^40,41^. This hypothesized mechanism would also explain why we observed more signatures of sleepiness during the self-driving condition relative to the manual condition even though we studied the same individuals for both drives. We found that SREMs were only related with prior sleep history in the self-driving condition when no music was present, suggesting that supervising an autonomous vehicle unmasks latent sleepiness in the absence of other stimulation. However, no individuals in the music study slept less than five hours per night, compared with a few individuals who had extremely short habitual sleep durations in Studies 1 and 2. This may explain why the overall number of SREMs were higher in the self-driving condition when individuals listened to music, but with no obvious relationship to prior sleep history.

We found that brain synchronization, but not spectral power was altered when individuals supervised a self-driving vehicle relative to manual driving. Delta power is a marker of the sleep homeostat that increases as sleepiness accumulates^42^. We did not observe differences in delta power between studies, which is not surprising given that we conducted a within-subjects study and the participants were studied on the same day (i.e., at approximately the same level of homeostatic sleep pressure). Using a novel metric to explore physiological markers of cognitive states, we observed reduced brain synchronization when participants supervised a self-driving vehicle. Specifically, we observed a reduction in the delta band for all three studies. This frequency band has been associated with fatigue, including driver fatigue^43^. Stated broadly, as a consequence of the macroscopic nature of the EEG signal, it is generally thought that higher frequency oscillations are the hallmark of local computation^44^, whilst lower frequency oscillations represent the integration and coordination of distal regions of the brain^45–47^. This long-range coordination, specifically within the delta band, has been implicated in decision making and has been purported to coordinate activity between the parietal and frontal cortex^48^. Coupled with literature that shows power fluctuations in the delta band related to motor control^49^, visual processing, and error signaling^50,51^, it seems likely that the synchronization effects are facilitating the complex decision and motor coordination in driving. Future studies are needed to disentangle these potential fatigue, homeostatic, and cognitive processes.

We also observed a significant difference within the alpha band synchronization across the brain, where supervising a self-driving car decreased brain synchronization, but not in Study 3 where individuals had attenuated SREMs while listening to music while driving. Research on the ubiquitous alpha band has suggested that EEG alpha activity may be involved in integrating and coordinating function across several cognitive systems^46,52^. Specifically, synchronization in the alpha band is related to a variety of cognitive phenomena with particular emphasis on the visual system^53^. Together with our behavioral results that show listening to music may attenuate the effects of sleepiness in the self-driving condition, our results in alpha band synchronization may also suggest an attenuation of visual information processing or attentional deficits as a consequence of the observed sleepiness in Study 1 and Study 2.

Our findings have important implications for other modes of transportation, such as in commercial trucking and aviation, as the prevalence of autonomous systems in these domains increases. Fatigue arising from sleep loss and circadian misalignment has long been a concern in these operations due to long work hours and 24-h work requirements^54,55^. In trucking, where long episodes of highway driving are often required at night, the risk of a driver failing to supervise a self-driving truck would likely increase relative to what we observed during a daytime drive. In aviation, autopilot is frequently used during the cruise portion of a flight, when workload and risk of an incident or accident are typically low. However, sensor failures guiding autonomous systems in aircraft, such as those that led to two recent accidents^56,57^, require expert knowledge and rapid response. As a result, it is critical that flight crew maintain situation awareness and skills to take control of the aircraft when necessary. Further research is needed in these domains to better inform how supervision of autonomous systems may influence these specialized operators and how interventions may protect individuals from expressing these behaviors.

Although we conducted three randomized trials to evaluate how sleepiness influences supervision of self-driving vehicles compared to manually controlled driving, our study is not without limitation. We studied individuals following their typical sleep schedules in order to evaluate how alertness and performance might be affected during autonomous and manually controlled driving under naturalistic conditions. Some of the individuals that we studied were relatively rested, while others were sleep deficient and some of the outcome measures that we examined vary greatly within an individual^8,58^. This may explain why individuals in Study 1 had more SREMs and slower reaction times during the self-driving condition compared to those in Study 2. However, we utilized a within-subjects design to help account for these effects. Further studies are needed to understand how different types of sleep loss (e.g., acute vs. chronic) influence one’s ability to supervise a self-driving vehicle. In addition, more study is needed to evaluate how other driving scenarios including inclement weather, traffic density, frequency of stops and lane changes, pedestrian traffic, different types of terrain (e.g., hills and curves), multi-passenger situations, and active engagement in non-driving activities influence driver attention. Such studies should provide insight to guide the development of targeted countermeasures to improve driver engagement. Our study did not require the driver to take control of the vehicle during the self-driving supervision task. As a result, it is unclear whether our findings would lead to failures to respond to such transitions of control. Finally, participants in our study completed an approximately 48-minute simulated drive. It is possible that longer or shorter drives in the real world may yield different outcomes.

In conclusion, we found that individuals following their typical sleep schedules experienced more SREMs, had their first SREM sooner and felt sleepier when supervising a self-driving vehicle relative to manually controlled driving, even while listening to music. Furthermore, we found that participants had more lapses of attention and slower response times following the self-driving supervision relative to manually controlled driving when driving without listening to music. These findings suggest that the risk of accidents could be higher with partially autonomous systems due to the rapid expression of sleepiness that arises from supervision of a self-driving vehicle. Further research is needed to understand how this sleepiness influences a driver’s ability to take control of the vehicle. Until the infrastructure is in place to allow for fully autonomous vehicles (i.e., level 5 automation with no driver input), mitigations must be developed to help sleepy drivers maintain situational awareness and to be ready to assume control of the vehicle when necessary.

## Methods

We conducted three separate simulated driving studies. The first study was conducted to evaluate sleepiness and vigilant attention among individuals while they supervised a self-driving vehicle compared to manually controlled driving. We conducted a second study to validate the findings of our first study. We conducted a third study to evaluate how listening to music while driving might alter attentional capacity while supervising a self-driving vehicle relative to manually controlled driving.

### Participants

Participants were required to be at least 18 years old with a valid driver’s license. Participants in Study 1 were required to have had a driver’s license for at least five years. We removed this restriction to increase the generalizability of our sample for Studies 2 and 3. There were no other exclusionary criteria, as we wanted to study a typical sample of drivers. None of the participants received financial compensation for the study, but four participants received extra credit in a statistics class for participating. All experimental protocols were approved by the NASA Human Research Institutional Review Board (HRIRB). All methods were carried out in accordance with relevant guidelines and regulations. All participants provided written informed consent prior to study participation (NASA HRIRB protocol # HRII-16–27 and HRII-17–34).

We based our power calculations on anticipated changes in reaction time comparing the self-driving to the manual driving condition. Based on previous studies in similar populations, we expected that the mean reaction time would be 300 ms with a standard deviation of 65 ms (due to individual variability in sleep history)^59^. Using these assumptions, we calculated that we needed 14 participants to detect a mean change of 50 ms with 80% power at an alpha level of 0.05. We requested permission to study up to 24 or 25 individuals in each study in order to account for individuals who were noncompliant or who withdrew consent. No participants withdrew from Study 1 (i.e., 19 individuals signed the consent form and completed the study). In Study 2, 24 individuals volunteered for the study and signed consent forms. Of these, four participants withdrew during the at-home data collection portion of the study; three withdrew due to scheduling conflicts and one withdrew after experiencing discomfort wearing the Actiwatch. In Study 3, 23 individuals volunteered for the study. Of these, three participants withdrew due to being unable to find a suitable day to schedule the laboratory visit.

Each study (Studies 1, 2, and 3) was comprised of a different set of volunteers, for a total of 59 unique participants who completed the laboratory study (Study 1, *n* = 19; Study 2, *n* = 20; Study 3, *n* = 20).

### Experimental design

#### Pre-study measures

During a study orientation, participants completed questionnaires describing their demographics and driving history, and completed the Horne-Ostberg Morningness-Eveningness Questionnaire (MEQ)^60^, the Fatigue Severity Scale (FSS)^61^, and the Pittsburgh Sleep Quality Index (PSQI)^62^.

To assess the relationship between sleep history and driving performance, participants wore an actigraphy monitor (Actiwatch Spectrum Pro or Plus, Philips-Respironics, Bend, OR, USA; set to one-minute epoch collection) on their non-dominant wrist and recorded their sleep and wake time in a sleep diary for two weeks prior to the driving study. Participants were told not to alter their typical sleep habits during the two weeks prior to the study.

#### Laboratory study

After collecting two weeks of sleep data, participants completed a single three-hour daytime laboratory visit in a dim, temperature constant, and sound-attenuated laboratory located at NASA Ames Research Center. Participants wore electrodes for the evaluation of brain activity and transitions to sleep while they completed two simulated driving sessions in a randomized order: one in the manually controlled driving condition in which participants drove an automatic transmission vehicle and had full control of the vehicle dynamics (i.e., steering, acceleration, brakes); and one in the self-driving (autonomous) condition in which participants had no control of the vehicle dynamics.

All participants in all three studies completed both the manually controlled and self-driving simulations. The order of these conditions was randomized in a counterbalanced design for each study in blocks of four, resulting in 10 participants randomized to start with the manually controlled driving condition in all three studies. Randomization occurred on the day of the laboratory visit, therefore participants who withdrew consent before participating in the laboratory visit were not randomized.

A researcher remained in the room during the driving session to ensure that the participants followed study procedures. The researcher did not interact with the participant once the driving session started unless the participant was non-compliant with the study protocol (e.g., removing hands from the wheel). Participants were not allowed to eat, drink, chew gum, or talk to the researcher during the study. After each driving session, participants rated their level of sleepiness using the Karolinska Sleepiness Scale (KSS)^63^ and completed a five-minute psychomotor vigilance task (PVT)^64^.

### Driving simulation

#### Equipment

The driving simulator consisted of a 32-inch TV display (Samsung) and an electronic steering wheel with gas and brake pedals (Logitech G27). SimCreator (RealTime Technologies, Ann Arbor, MI, USA) was used to construct the driving scenario and vehicle dynamics. Each simulation was identical.

#### Driving scenarios

The driving scenario consisted of a flat, monotonous, two-lane road in the countryside with no stop signs or traffic lights. The simulation included 14 curved segments in between straight segments (Supplemental Fig. 1). There were buildings and trees interspersed between open fields and there were 63 road signs along the route (e.g., speed limit signs, deer signs, curve signs). The simulation included light traffic with cars passing the simulation vehicle. The simulation also included three cases where a pedestrian crossed the road and three cases where a vehicle braked in front of the simulated vehicle. In such cases the driver would be required to slow down in the manually controlled drive and the self-driving vehicle would slow in response to the event without any user input. There were no situations in the self-driving condition where the participant was required to take over control of the vehicle.

For both of the driving conditions, participants were instructed to merge onto the right lane and remain in the right lane of the two-lane road, keep both hands on the steering wheel at all times, and adhere to the speed limit signs. In the self-driving scenario, the participant pressed a button on the steering wheel upon merging onto the highway to engage the automation. The participants oriented their hands so that they were at the “10” and “2” number positions on the face of the clock. In order to ensure that participants maintained task engagement during both conditions, we instructed participants to press the button on the steering wheel when they saw road signs that appeared on the right side of the road during each of their driving sessions.

The self-driving condition lasted 48 min. The manually controlled driving condition was on the same circuit, but the duration of the drive could have been slightly longer or shorter than the autonomous drive depending on the speed that the participant traveled while driving. Prior to the experimental driving sessions, participants completed a five-minute practice session in order to familiarize themselves with the vehicle controls (e.g., steering wheel, gas and brake pedals) and to ensure they understood the instructions.

### Music administration

Participants who completed the music study were asked to create their own music playlist prior to the drives by selecting a genre or artist from Spotify. They were then asked to review the playlist to remove any songs that they did not like before commencing the study as they were informed that they would not have access to the playlist (e.g., to skip songs) during the drives. The playlist was started at the beginning of each drive and was paused during the testing that occurred between the drives. There were no advertisements or interruptions to the music stream once it was started.

### Measures

#### Electroencephalography recording and slow rolling eye movement detection

Gold cup electrodes were used to measure electroencephalography (EEG) and electrooculography (EOG) activity and placed according to the International 10–20 system. Four electrodes (the z-line) were applied to the scalp at the frontal (Fz), central (Cz), parietal (Pz), and occipital (Oz) derivation. Additionally, one electrode was applied one centimeter below and one centimeter lateral to the outer canthus of the left eye, and another electrode was applied one centimeter above and one centimeter lateral to the outer canthus of the right eye. Electrode impedances were checked and confirmed as < 10 kOhms prior to the study. BrainVision Recorder (Brain Products GmbH, Munich, Germany) was used to record EEG and EOG during each driving session at a sampling rate of 500 Hz, high cutoff filter of 70 Hz, and notch filter of 60 Hz.

Prior to data analysis, BrainVision Analyzer 2 (Brain Products GmbH, Munich, Germany) was used to reference all of the electrodes to a single, averaged mastoid reference channel (A1 and A2). All physiological signals were high-pass (0.4823 Hz, time constant: 0.33 s, 12 dB/octave) and low-pass filtered (70 Hz, 24 dB/octave) offline.

An automated method was devised to carefully remove time segments that contained any eye movements for the assessment of brain activity. All preprocessing, artifact editing, and EEG analysis was completed in Matlab (Mathworks, Inc.). For this process, we (1) estimated the magnitude of the product of the EOG channel traces and used this as a marker for eye movements and blinks, and then (2) removed the two seconds centered around amplitude fluctuations larger than three standard deviations of the mean of product of the signal estimated in step 1. On average, this resulted in 16.5% of the data removed (SD = 13.9%). After this cleaning, we next analyzed the EEG signals in two ways. First, we estimated the average power spectral density across channels using a traditional approach that robustly measures power of an input signal – Welch's averaged modified periodogram method of spectral estimation^65^. Next, we derived connectivity across the electrodes by estimating synchronization via the binarized derivative method (BDM)^14^, which is a computationally inexpensive way to estimate the proportion of input signals whose increases and decreases in power fluctuations are consistent across time. For this metric, we modified the traditional BDM slightly to estimate band-specific synchronization. In brief, this method consists of five steps after preprocessing: (1) narrow band filtering of the preprocessed signals into common frequency bands of interest (delta: 1–3 Hz, theta: 4–7 Hz, alpha: 8–12 Hz, beta: 13–25 Hz, gamma: 26–40 Hz); (2) estimating the power fluctuations across time via a Hilbert transform; (3) estimating the first derivative for each of the four electrodes; (4) converting the first derivative signal into 1 or -1 via a signum function to indicate an increase or decrease of power at each time point; and (5) finally calculating the average sum of the processed signals across time, which is what we define as *synchrony* here. Thus, this rapid derivative method results in a value that signifies the propensity of the envelope of power fluctuations in the electrodes to dynamically change synchronously.

The EEG records were also visually inspected by a blinded scorer in 30-s epochs (EEFE). Slow rolling eye movements (SREMs) were marked and were defined as out-of-phase, rolling deflections lasting at least 0.5 s in both channels of the EOG. SREMs are a feature of the transition to stage 1 sleep^12^.

#### Psychomotor vigilance task (PVT)

A five-minute version of the psychomotor vigilance task (PVT) was administered on an iPod (6th generation, Apple, Cupertino, CA, USA)^64^ following each drive. The PVT is an established measure of vigilant attention^8^. The primary outcome measures were mean reaction time and lapses of attention, which were defined as reaction times > 500 ms. The mean reaction time was adjusted by the system latency of 82 milliseconds^66^, then mean reaction time and number of lapses were computed for each participant for each condition.

#### Actigraphy

Participants’ bedtime and waketime from their sleep diary were inputted as rest intervals into Philips Actiware (version 6.0.9) for analysis using a medium wake threshold value of 40 activity counts and a sleep onset/end setting of 10 immobile minutes. Bedtime was defined as the time when the participant went to bed with the intent to sleep and waketime was defined as the time when the participant rose from bed for the final time. The sleep duration for each day was calculated by summing the number of minutes categorized as sleep during their main sleep episode and any nap intervals the following day. The mean sleep duration for each participant was calculated by averaging the sleep duration of the 14 days prior to the laboratory study. Two participants did not return their sleep diary and thus their estimated bedtime was based on the first instance in which the activity count fell below 150 and the epoch status was categorized as “sleep,” and their estimated waketime was based on the first instance in which the activity count was above 150 and the interval status was categorized as “active”. This method was also used in instances where participants forgot to record their bedtime and waketime for any sleep episode.

### Statistical analysis

All analyses were calculated using SAS Software (Version 9.4, Cary, NC) and Matlab (Mathworks, Inc). Univariate summary statistics were calculated for all variables of interest. Statistical significance was set at α < 0.05 for all tests.

The number of SREMs experienced by each participant were counted and compared between conditions. Count data is discrete, positive, and often includes excess 0 values, which makes it inappropriate to apply linear models to the data. We evaluated our data and selected a negative binomial regression model because many individuals experienced few SREMs and the data were overdispersed (i.e., the variance was greater than the mean). We adjusted the model to account for the within-subjects correlation (proc genmod, with subject as a repeated factor). Negative binomial models were also used to compare SREMs to average sleep duration by study and condition. *χ*^2^ values were computed using the “estimate” function in proc genmod.

Comparisons of time-to-first-SREM during each drive were conducted using log-rank tests, adjusting for the correlation between participants (proc lifetest). All participants were included in this analysis, but data for three participants were censored (one in Study 2 due to a software failure and two in Study 3 due to a software failure and a fire alarm; each of these incidents caused the second drive to end prematurely). A software error occurred for other participants, but SREMs occurred before the software failure, which allowed the inclusion of those data.

The KSS is a 9-point Likert scale. As this type of data is not normally distributed, differences between the KSS ratings following each drive were computed and compared using the non-parametric Wilcoxon signed-rank test.

PVT reaction time was assessed using linear mixed effects models (proc mixed in SAS), with subject and intercept included as random effects, with unstructured covariances. Counts of PVT lapses between conditions were compared using negative binomial models because the data were overdispersed. These models were adjusted for the within-subjects correlation (proc genmod, with subject as a repeated factor).

EEG synchronization was assessed using the fitlme.m function within Matlab (Mathworks, Inc), with subject and intercept included as random effects and unstructured covariances, mirroring the analysis completed on the PVT reaction times. Additional analyses of the EEG were conducted using paired-samples *t*-tests in Matlab with the ttest.m function.

Effect sizes for all comparisons were calculated using *Hedge's g* and were adjusted to reduce sample size bias as follows:

$$g = \frac{{M_{{1~}} - M_{2} }}{{SD_{{pooled}}^{*} }} \times \left( {\frac{{N - 3}}{{N - 2.25}}} \right) \times \sqrt {\frac{{N - 2}}{N}}$$

SREMs, KSS ratings, and PVT reaction time and lapses were calculated for each participant and each drive and compared. A software error caused premature termination of the second drive for two participants in Study 1, and for one participant in Study 2. In addition, in Study 3, the EEG software froze for five minutes in the middle of the drive for one participant (in this case the participant completed the drive without incident) and there was a fire alarm incident that required termination of the study approximately seven minutes into the second drive for one other participant. The data from these participants were excluded from mean SREM comparisons (Study 1 *n* = 17; Study 2 *n* = 19; Study 3 *n* = 18). These data were also excluded for KSS comparisons, except for the participant in Study 3 who experienced the EEG software error but completed both drives (Study 1 *n* = 17; Study 2 *n* = 19; Study 3 *n* = 19). These data were also excluded for PVT comparisons, except for the participant in Study 3 who experienced the EEG software error. In addition, an administrative error in Study 1 led to the loss of PVT data for eight participants (Study 1 *n* = 9; Study 2 *n* = 19; Study 3 *n* = 19). All data were included in log-rank tests with censoring as described above (Study 1 *n* = 19; Study 2 *n* = 20; Study 3 *n* = 20).

### Data and code availability

All data and code associated with this manuscript will be housed at NASA Ames Research Center and will be available upon request following review and approval from the Principal Investigator and NASA Institutional Review Board.

## Supplementary Information

Supplementary Information.
