# Supplementary material for: Supervision of a self-driving vehicle unmasks latent sleepiness relative to manually controlled driving
Source: Sci Rep. 2021 Sep 14;11:18530. doi: 10.1038/s41598-021-92914-5 (PMC8440771; doi:10.1038/s41598-021-92914-5)
Supplement: Supplementary file 1 — Supplementary Information. [file 41598_2021_92914_MOESM1_ESM.pdf]

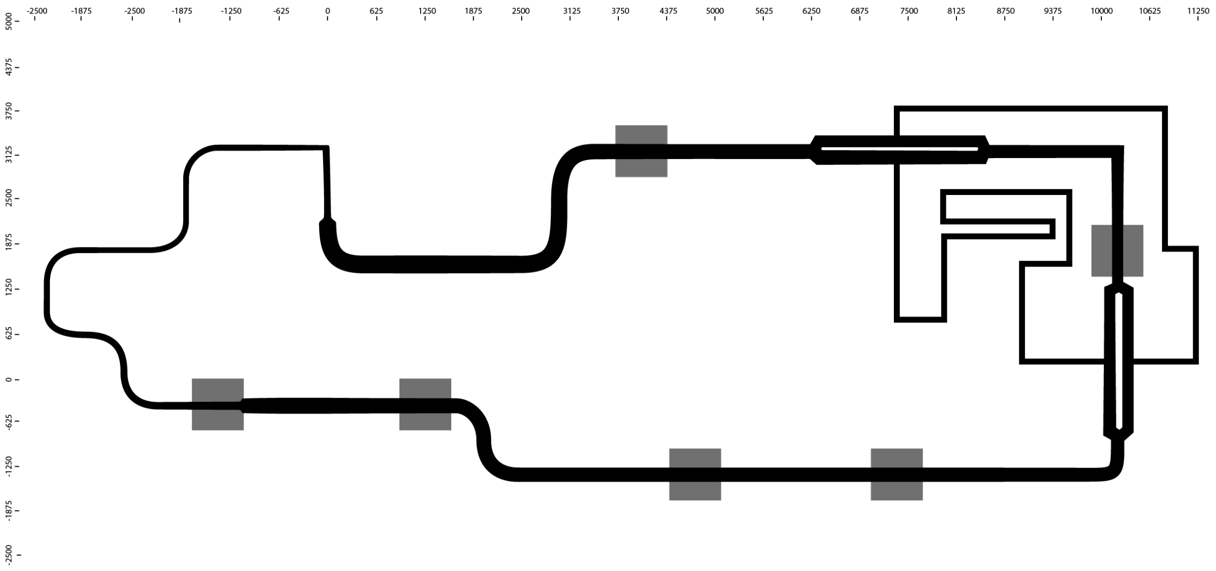

Supplemental Figure 1. Schematic of the driving circuit. Thick black lines represent the driving circuit. Gray boxes represent bridges. The thickness of the line reflects the width of the highway. The speed limit was reduced in areas where the road was narrower. The thin black lines on the right side of the schematic represent other roads that were not used during this simulation.
